# Correction: Alamandine attenuates hepatic fibrosis by regulating autophagy induced by NOX4-dependent ROS

**DOI:** 10.1042/CS-20191235_COR

**Published:** 2021-12-15

**Keywords:** Alamandine, Angiotensin II, Hepatic fibrosis, NADPH oxidase, Oxidative stress

This Correction follows an Expression of Concern relating to this article previously published by Portland Press.

The authors of the original article “Alamandine attenuates hepatic fibrosis by regulating autophagy induced by NOX4-dependent ROS” would like to address concerns raised by a reader regarding Figure 3B,J in their published paper. The authors have acknowledged that an incorrect figure was used by mistake in the preparation of the manuscript. Upon further investigation, the authors have identified errors in Figure 1F, Figures 3B,J,4B,5B,D in the originally published paper and would like to address these errors with this Correction and the provision of corrected figures. The authors would like to apologize to readers for these errors, stating there is no intentional concealment such as intentionally modifying the images or flipping the images. All incorrect images have been corrected. The corresponding original experimental images are also provided, please refer to the raw data (Supplementary Material). Please also note the correction of the affiliation for Ying Meng: Department of Respiratory Diseases, Nanfang Hospital, Southern Medical University, Guangzhou 510515, China.

**Figure 3:**
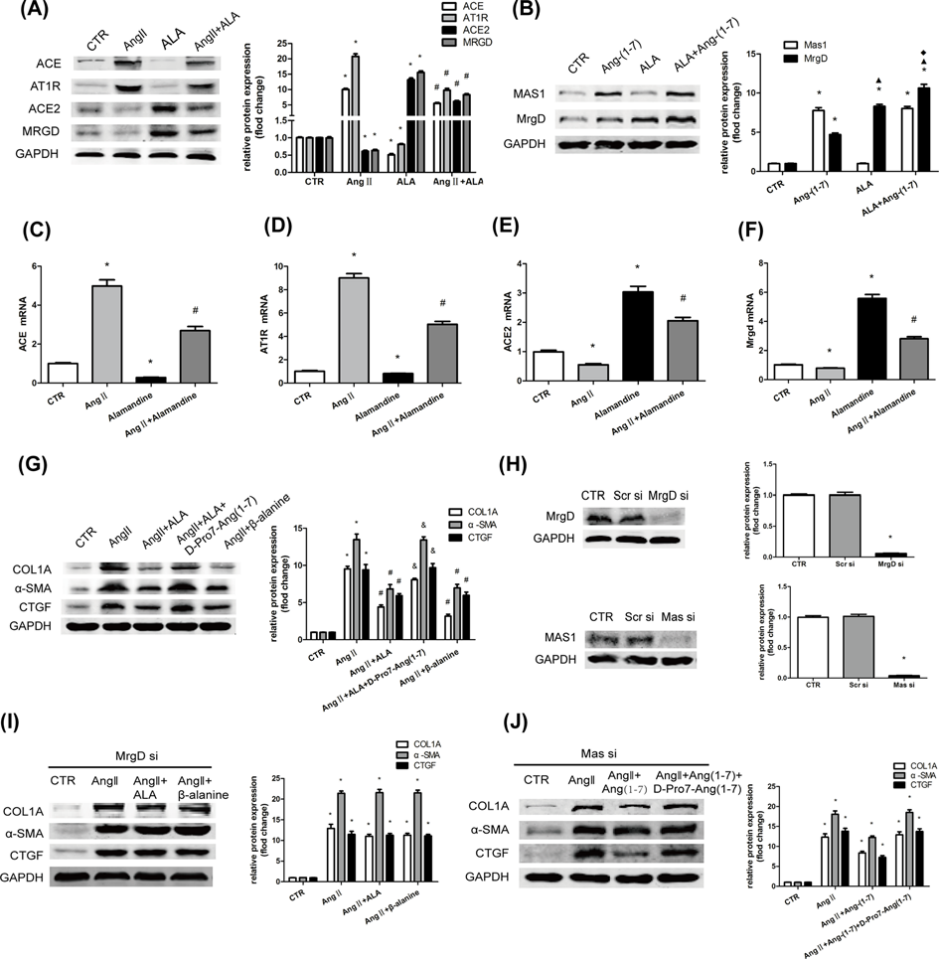
Alamandine exerts anti-fibrotic actions via MrgD receptor in HSCs along with up-regulation of the ACE2/alamandine/MrgD axis (**A**) HSCs were pretreated with ALA (10^−7^ M) for 1 h before stimulation with Ang II (10^−7^ M) for 24 h. Protein levels of ACE, AT1R, ACE2, and MrgD were measured by Western blot analysis. (**B**) HSCs were pretreated with ALA (10^−7^ M) or/and Ang-(1–7) (10^−7^ M) for 24 h. Protein levels of MAS1 and MrgD were measured by Western blot analysis. (**C-F**) The mRNA levels of ACE, AT1R, ACE2, and MrgD of HSCs with various treatments were determined by qRT-PCR. (**G**) HSCs were pretreated with ALA (10^−7^ M), D-Pro^7^-Ang-(1–7) (10^−5^ M), or β−alanine (10^−7^ M) for 1 h before stimulation with Ang II (10^−7^ M) for 24 h. Protein levels of COL1A, a-SMA, and CTGF were measured by Western blot analysis. (**H**) HSCs were transfected with MrgD siRNA or Mas siRNA, protein levels of MrgD or Mas1 was measured by Western blot analysis. (**I,J**) The HSCs, which had been interfered with MrgD siRNA or Mas siRNA, were pretreated with ALA (10^−7^ M), Ang-(1–7) (10^−7^ M), or β-alanine (10^−7^ M) for 1 h before stimulation with Ang II (10^−7^ M) for 24 h. Protein levels of COL1A, α-SMA, and CTGF were measured by Western blot analysis. All of the assays were performed in triplicate. The data are presented as mean ± SEM. *P<0.05 versus CTR; ^#^*P*<0.05 versus the Ang II treatment group; ^&^*P*<0.05 versus the Ang II + ALA treatment group; ^▲^*P*<0.05 versus the Ang-(1–7) treatment group; ^♦^*P*<0.05 versus the ALA treatment group. Abbreviation: CTR, control.

**Figure 4:**
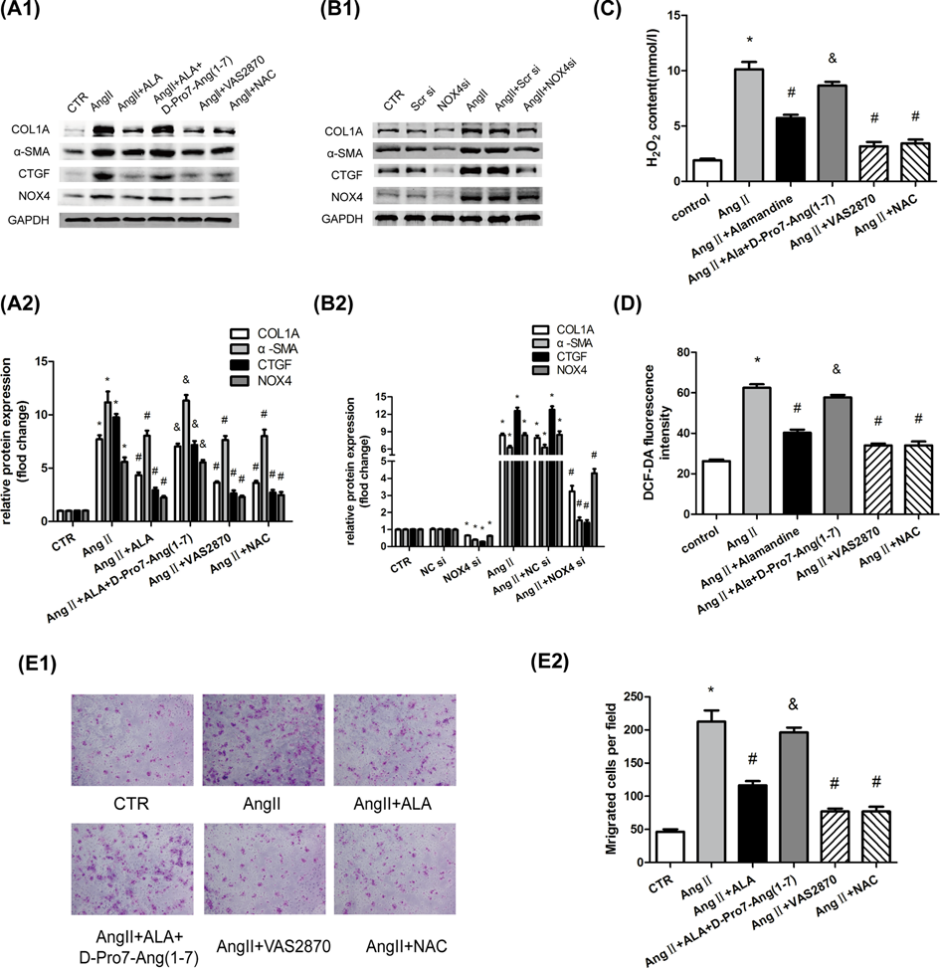
Alamandine suppresses Ang II-induced collagen production by inhibiting oxidative stress in HSCs HSCs were pretreated with alamandine (10^−7^ M), VAS2870 (10^−5^ M), or NAC (10^−3^ M) for 1 h before stimulation with Ang II (10^−7^ M) for 24 h. HSCs were pretreated with D-Pro^7^-Ang-(1–7) (10^−5^ M) for 1 h before stimulation with alamandine (10^−7^ M). (**A**) Protein levels of COL1A, α-SMA, CTGF, and NOX4 were measured by Western blot analysis. (**B,C**) HSCs were pretransfected with NOX4 siRNA or MrgD siRNA before stimulation with Ang II (10^−7^ M) for 24 h. The protein levels of COL1A, α-SMA, CTGF, and NOX4 were measured by Western blot analysis. (**D**) The H_2_O_2_ concentration in HSCs was measured. (**E**) Intracellular ROS was detected by the probe DCF-DA. (**F**) Cell motility was detected by cell migration assays. All the assays were performed in triplicate. The data are presented as mean ± SEM. *P<0.05 versus CTR; ^#^*P*<0.05 versus the Ang II treatment group; ^&^*P*<0.05 versus the Ang II +ALA treatment group. Abbreviation: CTR, control.

**Figure 5:**
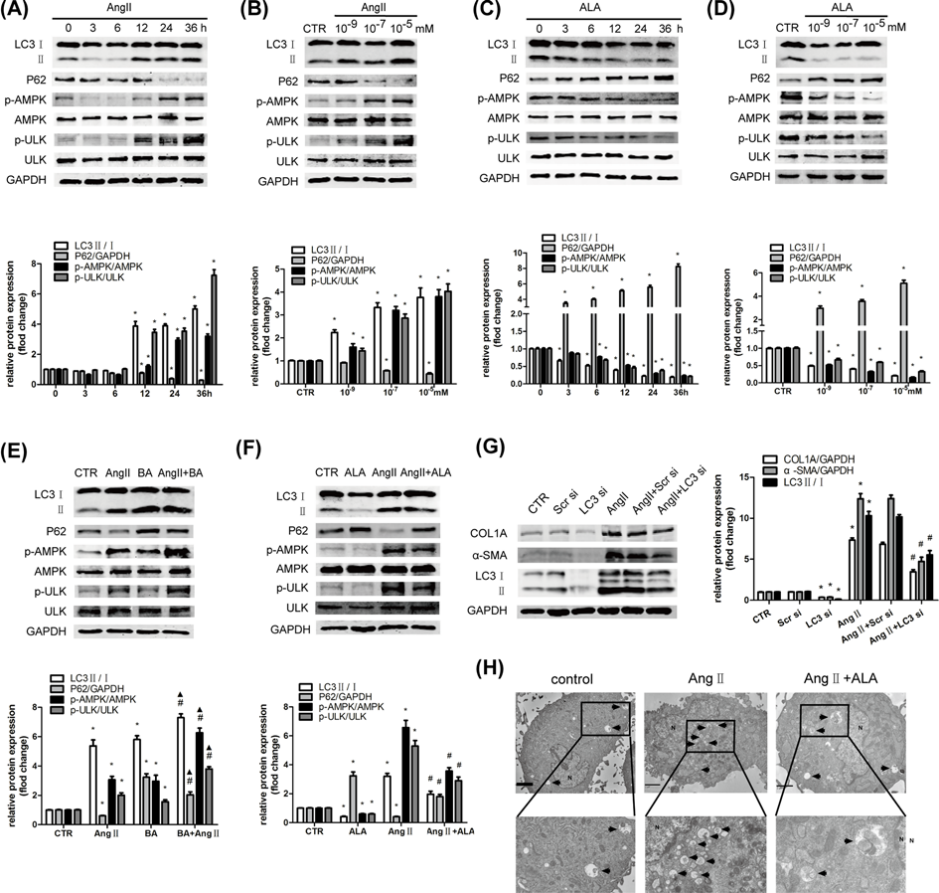
Alamandine attenuates the AMPK-dependent autophagy in HSCs (**A-D**) HSCs were treated with varying times of Ang II (10^−7^ M) or alamandine (10^−7^ M) or varying concentrations of Ang II or Ang-(1–7) for 12 h. The protein levels of AMPK, ULK1, P62, and LC3 II/I were analyzed by Western blot. (**E,F**) HSCs were pretreated with BA (10 nM) or alamandine (10^−7^ M) for 1 h before stimulation with Ang II (10^−7^ M) for 24 h. The protein levels of AMPK, ULK1, P62, and LC3 II/I were analyzed by Western blot. (**G**) HSCs were pretransfected with LC3 siRNA before stimulation with Ang II (10^−7^ M) for 24 h. The protein levels of COL1A, α-SMA, and CTGF were analyzed by Western blot. (**H**) Autophagosomes structures (denoted by black triangles) in HSCs with a high-magnification transmission electron microscope (scale bar: 2 µm). All the assays were performed in triplicate. The data are presented as mean ± SEM. *P<0.05 versus CTR; ^#^*P*<0.05 versus the Ang II treatment group; ^▲^*P*<0.05 versus the BA treatment group. Abbreviations: CTR, control.

## Supplementary Material

Supplementary Material

