# Supplementary material for: Correction: Alamandine attenuates hepatic fibrosis by regulating autophagy induced by NOX4-dependent ROS
Source: Clin Sci (Lond). 2021 Dec 15;135(24):2665. doi: 10.1042/CS-20191235_COR (PMC8674567; doi:10.1042/CS-20191235_COR)

fig 3B

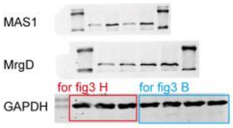

Special explanation about GAPDH of Fig3 B:
This original image of the internal reference image contains two independent experiments in this article.

fig 3J

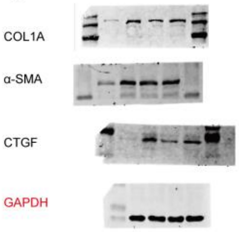

fig 1F

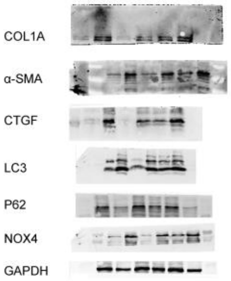

fig 4B

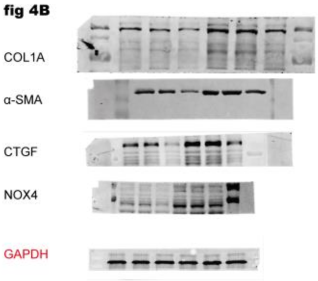

fig 5B

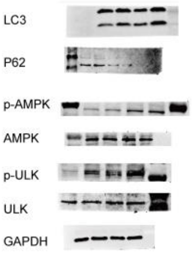

fig 5D

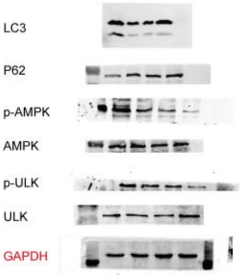

Supplement: Supplementary Material [file CS-2019-1235_COR_supp.pdf]
